# Envirotyping for deciphering environmental impacts on crop plants

**DOI:** 10.1007/s00122-016-2691-5

**Published:** 2016-03-01

**Authors:** Yunbi Xu

**Affiliations:** Institute of Crop Science, Chinese Academy of Agricultural Sciences, 100081 Beijing, China; International Maize and Wheat Improvement Center (CIMMYT), El Batan, Texcoco, CP 56130 Mexico

## Abstract

Global climate change imposes increasing impacts on our environments and crop production. To decipher environmental impacts on crop plants, the concept “envirotyping” is proposed, as a third “typing” technology, complementing with genotyping and phenotyping. Environmental factors can be collected through multiple environmental trials, geographic and soil information systems, measurement of soil and canopy properties, and evaluation of companion organisms. Envirotyping contributes to crop modeling and phenotype prediction through its functional components, including genotype-by-environment interaction (GEI), genes responsive to environmental signals, biotic and abiotic stresses, and integrative phenotyping. Envirotyping, driven by information and support systems, has a wide range of applications, including environmental characterization, GEI analysis, phenotype prediction, near-iso-environment construction, agronomic genomics, precision agriculture and breeding, and development of a four-dimensional profile of crop science involving genotype (*G*), phenotype (*P*), envirotype (*E*) and time (*T*) (developmental stage). In the future, envirotyping needs to zoom into specific experimental plots and individual plants, along with the development of high-throughput and precision envirotyping platforms, to integrate genotypic, phenotypic and envirotypic information for establishing a high-efficient precision breeding and sustainable crop production system based on deciphered environmental impacts.

## Introduction

Climate change has resulted in significant changes in weather pattern, precipitation distribution, temperature and moisture fluctuation, soil erosion, and desertification (FAO 2008), although the average annual environmental measurements may not change significantly. Extreme conditions caused by these changes bring about many unexpected and more frequent biotic and abiotic stresses (Bebber et al. 2013; Trenberth et al. 2014). To feed increasing world population, total crop production will need to be significantly increased with less arable land under much severe environmental conditions (Tilman et al. 2002). For the past 50 years, such demanding has been met by continuous yield improvement. Unfortunately, yield growth has been slowing, rather than increasing as required by global population increase. For example, annual yield growth for three major cereals, rice, wheat and maize, has been decreased to 0.79–1.74 % for 1990–2010 from 2.19–2.95 % for 1960–1990. Moreover, if such reduction tendency continues, yield growth for 2010–2050 will decrease to 0.62–1.33 % (FAO 2014; Pardey et al. 2014). For the next 50 years, we will have more people, but less water on the planet, and have to develop two times better crops for a world free of poor, poverty, and environment degradation. To meet the challenges, we need to keep enhancing yield potential while filling the yield gap created by various abiotic and biotic stresses largely caused by climate change. Therefore, environmental factors that affect plant growth and yield should be understood and managed better for less degradation and input but more output.

Crop production has been largely affected by environmental factors that affect all the processes from metabolism to gene expression during plant growth and development. Increasing yield and filling yield gap largely depend on the management, control and improvement of the environments where crop plants grow. The genotypes (*G*) that determine the yield potential and their responses to environmental factors can be now investigated and measured through molecular and genomic approaches using chip or microarray (Hoheisel 2006) and sequencing technologies (Koboldt et al. 2013). The phenotypes (*P*) can be also measured precisely with the development of high-throughput phenotyping tools and methodologies (Araus and Cairns 2014). Compared with genotyping and phenotyping, determination and measurement of environmental factors (*E*) has fallen behind, largely due to three reasons. First, environmental factors have been largely considered as a whole and treated as a blackbox that interacts with genotypes to affect plant growth and yield, without dissection for individual plants. Second, only major environment factors have been considered and measured at the level of the whole experiment station or trial. Third, most environmental factors are dynamic and constantly changing throughout the plant growing period. Dissection of quantitative traits into individual Mendelian factors using molecular markers allows quantitative genetics walk out of the multiple-gene circle taking all the relevant genes as a whole (Paterson et al. 1988; Lander and Botstein 1989). A similar significant impact would be made if complex environments could be partitioned into individual factors and measured for individual plants and every developmental stage.

Understanding better the environment where plants live is critical to our future crop science for several reasons. Firstly, gene expression is largely dependent on the environment where the crop grows. Secondly, genetic mapping and gene cloning depends on the environment where the phenotyping is performed. Thirdly, many phenotyping procedures, including abiotic and biotic stress evaluation, is conducted under managed environmental conditions. Lastly, environmental assay becomes increasingly important for many procedures of crop production. Precise dissection of complex environmental factors for both target environments and specific genotypes provides us a novel opportunity for management, control and optimization of environmental factors for enhanced genetic improvement and more efficient crop production. All environmental factors that affect plant growth and yield can be defined as envirotypes (environment + types). The process for determination and measurement of all the environmental factors is called envirotyping (Xu 2015). The concept was first proposed at two international conferences as “etyping” (Xu 2011, 2012), followed by journal articles with more details (Xu et al. 2012; Xu 2015). The term “envirotyping” has also been used recently by other researchers to refer to the collective body of methodologies that are applied to characterize environments within multiple environmental trials and the frequent repeatable environment types within the target population of environments (Cooper et al. 2014, 2016). Envirotyping is different from conventional environmental assay in three aspects. First, envirotyping will measure all environmental factors that affect plant growth and production instead of only for the major ones. Second, envirotyping will zoom into specific field plots and individual plants so that the envirotypic data will be collected to match up with the corresponding genotypic and phenotypic data. Third, crop management and companion organisms will be included as a part of environmental factors so that their effects on crop plants can be investigated. As a new concept, envirotyping will be fully discussed in this article, including its conception, implementation, and application in crop science, by which environmental impacts on crop plants can be deciphered.

## Environmental variables and envirotyping

### Environmental variables

Environmental factors can be micro- or macro-, non-organic or organic, and internal or external. Plant growth and yield are coordinated by both intercellular and external environments. Intercellular environments in plants are largely dominated by what are essentially enclosed in vacuoles, which consist of water (inorganic and organic molecules), waste products and small molecules with internal hydrostatic pressure or turgor, temperature, and an acidic pH maintained. The internal environments are largely affected due to the changes of pH, osmotic pressure and temperature, etc., caused by material exchange and signal transduction with external environments. Through a series of receptors, signal transductions and responses, plants make full responses to specific external environmental factors, resulting in ion transmembrane transport, metabolic pathway regulation, cytoskeleton modification and gene expression regulation (Nicotra et al. 2010).

External environmental factors can be classified into four categories, climate, soil factors, biotic factors, and crop management or cropping system (Table 1). Climate factors, such as temperature, radiation, precipitation or water availability and wind, determine where a plant can grow, while other factors determine how a plant grows. Some companion organisms, such as pathogens, pests and weeds, cause damages or stresses to the plants, while others, such as azotobacteria, are beneficial. Crop management, as a unique environment component, involves intercropping, rotating and agronomic practices. Environmental factors that affect plant growth and yield can be modified or dramatically changed by human activities. Controlled or artificial environments can be created using growth chambers, phytotrons, hydroponic or other manmade facilities. Crop production activities per se have contributed to some significant environmental changes such as fertility depletion, air pollution, acid rain, water contamination (toxic element accumulation), noise (dynamic disturbance), salinity, land weathering, and desertification. Climate change and globe warming may result in extreme environments and wild fluctuation of environmental factors, which impose severe stresses on plants, including biotic stresses caused by companion organisms and abiotic stresses associated with climate and soil factors. For survival and sustainable production, crop plants must cope with all the challenges from climate change catastrophes, including stressful water regimes, extreme temperatures, elevated CO_2_ and salinity, which impact on all aspects of plant architecture individually or in combination (Ahuja et al. 2010). Expanding of human population needs to increasingly explore less-farmable land with significant abiotic stresses, particularly for the pool soils with abnormal pH, low fertility, and salinity stress (Masuka et al. 2012). As a major constraint to crop yield in tropical regions, poor and depleted soil fertility force farmers into marginal lands and non-farming areas (Pingali and Pandey 2000). To stabilize crop production, stresses caused by pathogens, pests and other companion or symbiontic organisms should be paid more attention, as they are less predictable than soil or climate stresses. On the other hand, multiple abiotic stresses become increasingly prevalent. For example, heat stress often happens with water deficiency, while drought is accompanied by salinity (Ahuja et al. 2010). Abiotic and biotic stresses may occur simultaneously (Bostock et al. 2014; Kissoudis et al. 2014; Ramegowda and Senthil-Kumar 2015; Prasch and Sonnewald 2015), and one stress may show positive or negative impact over the other.

**Table 1:** External environmental factors affecting plant growth and yield

Category	Description	Effects and associated stresses
Climate factors		
Light	Solar radiation, light intensity (elevation, latitude, and season; clouds, dust, smoke, fog and smog), day length (photoperiod)	Most crucial factor for plant growth and development; shading stress
Temperature	Effective accumulated temperature; average, minimum and maximum daily temperatures	Photosynthesis, water and nutrient absorption, transpiration, respiration and enzyme activity, germination, flowering, pollen viability, fruit/seed set, rates of maturation and senescence, yield, quality, harvest duration and shelf life; cold, frost, and heat stresses
Water	Precipitation (rainfall, snow, hail, fog and dew)	Crop productivity and quality; drought, flooding and waterlogging stresses
	Atmospheric humidity (relative humidity)	Soil evaporation and plant transpiration; dry air stress
Air	Wind velocity	Supply of moisture, heat, and fresh CO_2_; strong wind stress
	Atmospheric gases (CO_2_, O_2_, N); pollutants (SO_2_, CO, CH_4_)	Air pollution and shading stresses
Soil factors		
Soil type	Soil type (clay, clayey loam, loam, sandy loam, and sand)	Soil’s capacity to store water and nutrients, aeration, drainage, and ease of field operations; soil-related stresses
Soil structure	Soil structure (texture, soil sealing, erosion, contamination, compaction, hydro-geological risks)	Crop productivity and quality contributed by soil fertility, organic matter and soil biodiversity; soil-borne stresses
Soil components	Soil moisture	Crop productivity and quality; drought, flooding and waterlogging stresses
	Soil air	Water absorption, respiration of roots and micro organisms, nutrient availability, decomposition of organic matter; soil air stresses including O_2_ limitation
	Soil temperature	Soil physical and chemical processes, absorption of water and nutrients, germination of seeds and growth rate, microbial activity and processes in the nutrient availability; cold and heat soil stresses
	Soil pH	Nutrient availability and microorganism activities; acidic, saline and alkaline soil stresses
	Soil fertility (N, P, K, micronutrients/mineral and soil organic matters)	Plant nutrients and their balance for plant growth; nutrient deficiency stresses and nutrient use efficiency
	Soil salinity (electrical conductivity)	Osmotic tension and water takeup; salinity stress
Biotic factors		
Companion animals	Soil fauna (protozoa, nematode, snails, and insects)	Decomposition of raw organic matter, fixation of atmospheric nitrogen; damages to plant roots and other parts
	Animals around plants (pest insects, parasites, fungi, bacteria, viruses, predators, honey bees, wasps, human)	Cross-pollination and increasing yield, damage to crop yield; various abiotic stresses
Companion plants	Weeds, epiphytic and allelopathic plants	Competition for space, water, light and nutrients, mutual benefit (synergistic effect), interference with crop plants, releasing compounds, volatilization or decomposition of plant residues, inhibition or prevention of plant growth; various biotic stresses
Cropping system		
Intercropping	Companion crop(s)	Competition for space, water, light and nutrients, buffering and mutual benefit (synergistic effect); various biotic stresses
Rotating cropping	Fore-rotating crop(s)	Residual effects of agronomic practices from the fore-rotating crop; various biotic stresses

### Multiple environmental trial data

**Fig. 1:**
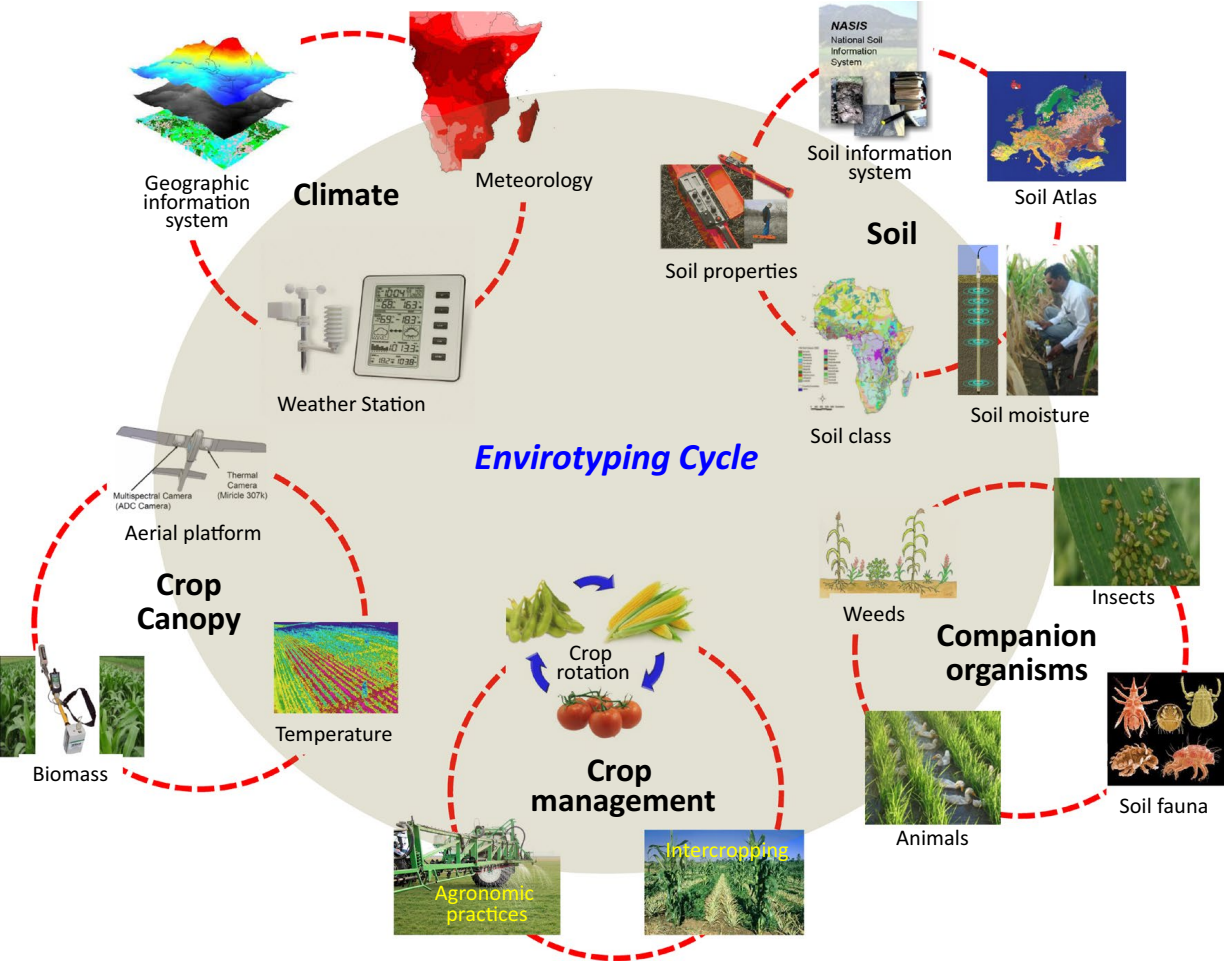
Envirotyping cycle. Environmental factors can be classified into five major groups, climate, soil, crop canopy, crop management and companion organisms, each containing several subgroups that describe important environmental factors affecting plant growth and development. Photos used for illustration were selected from public websites

Envirotyping can be implemented with a large amount of environmental information accumulated in crop science and production (Fig. 1). Multiple environmental trials (METs), involving a large number of genotypes tested in multiple locations, each with multiple replications, for multiple years (Johnson et al. 1955), can be considered a basic type of envirotyping for systematic collection of environment-related data. To identify the environments best suitable for commercialization of the on-trial varieties (genotypes), weather, climate and soil data have been collected systematically along with records of crop management including fertilization and control of diseases, pests, and weeds. In developed countries, such practices have been for decades. As an average effect of the environment, the empirical mean response for the *i*th genotype can be measured in the *j*th environment with *r* replications. Under Consultative Group on International Agricultural Research (CGIAR), International Maize and Wheat Improvement Center (CIMMYT) and International Rice Research Institutes (IRRI), for example, have implemented international breeding programs yield testing for many years, with a large amount of environmental data collected for three major crops, rice, wheat and maize. Such effort has been expanded to more countries and trial sites in recent years. However, envirotyping has not been well conducted in METs for three reasons: locations of MET sites are not precisely determined as needed; daily climate data linked to the trial sites are not available or difficult to collect; and data collection and completeness vary a lot across the sites.

### Geographic data

Geographic information system (GIS) has been established with the merging of cartography, statistical analysis and database technology, which is designed for collecting, storing, integrating, analyzing, and managing all types of geographical data (Fig. 1). The data for any location in Earth space–time can be collected as dates/times of occurrence, with longitude, latitude, and elevation determined by *x*, *y*, and *z* coordinates, respectively. GIS integrates various data sources with existing maps and up-to-date records from climate satellites. To capture climate data, various types of weather observatory stations have been established worldwide, including ground, radiosonde, wind, rocket, radiation, agrometeorological, and automatic weather stations. These stations document climate data for numerous locations and sites, which are transferred in international or national central databases and become a part of GIS data.

### Data from soil information systems

Soil data have been accumulating in worldwide soil information systems (Fig. 1). International Soil Reference and Information Centre (ISRIC) provides the international community with the world soil information. With a worldwide collaboration in soil data, soil mapping and their applications in global development issues, a centralized and user-focused World Soil Database is being developed, by which users can extract all validated and authorized data, including soil profiles and area-class soil maps (http://www.isric.org). To help bridge the soil information gap on the African continent, ISRIC has produced predicted information for various soil properties for the whole African continent at 250 m spatial resolution with multiple standard soil depths (http://www.isric.org). European Soil Portal (http://eusoils.jrc.ec.europa.eu/) is the focal point for soil data, contributing to a thematic data infrastructure with data and information regarding soils at European level, including maps and Atlases. At the national level, National Soil Information System (NASIS), USA, is one of the most comprehensive national systems, providing a dynamic resource of soil information for a wide range of needs. Soil Information System of China (SISChina) has been established to include soil spatial and attribute data, and China 1:1,000,000 soil database (Shi et al. 2007).

Lack of consistent soil classification systems across countries or organizations has hindered the communication and organizational functions. To bridge the gap, translations between systems should be developed. As a soil classification system for naming soils and creating soil map legends, the World Reference Base for Soil Resources (WRB) with its third edition has been released (IUSS Working Group WRB 2014), as an adopted system for soil correlation and international communication. It allocates every soil into one of the 32 Reference Soil Groups and then characterizes further each soil by a set of qualifiers. Information on the named soil, such as its genesis, ecological function and properties, will be provided through the system. To provide comprehensive spatial information, the same system can be refined slightly, and used to name the units of soil map legends. By accommodating national soil classification systems, WRB facilitates the worldwide correlation of soil information.

### Soil properties

Agricultural soils can be classified based on their physical texture, the size of the particles that make up the soil. Based on the particle-size distribution, soil texture can be further clarified into sand, silt and clay. Then crop suitability can be determined for each soil class, and the soil responses to environmental stresses and agronomic practices, such as drought or nutrient requirements, can be explored.

Currently available and potentially useful techniques for proximal, on-the-go monitoring of important soil physical properties (Whelan and Taylor 2013) can be used to measure soil texture/type, soil water storage capacity, soil water in season and waterlogging. In general, soil conductivity can be measured as a product of soil composition and formation. A typical “spread” of soil types gives a certain range of conductivity and resistivity, which match up with different soil types from sand to saline (Bevan 1998). Apparent soil electrical conductivity (ECa) measurement has been improved and becomes a widely accepted means to determine several soil physico-chemical properties (Corwin and Lesch 2005). Ground-based remote sensing technology has brought out a series of instruments for measurement of soil properties. For example, EM38, designed particularly for agricultural surveys and soil salinity measurement, provides a quick survey over large areas at depths of 1.5 and 0.75 m with its vertical and horizontal dipole modes, respectively. In a recent report on maize, measuring soil water content to 300 cm depth identified significant water extraction to a depth of 240–300 cm (Reyes et al. 2016), indicating that in-depth measurement of soil properties is required to capture a full profile of available resources for crops with large plant and root sizes such as maize.

Time domain reflectometry (TDR) systems, designed to detect cable breaks, are now widely used to determine soil water content, bulk electrical conductivity, and rock mass deformation. To monitor soil water profiles, PR2 soil moisture probe measures soil moisture at 4–6 depths down to 40–100 cm (http://www.delta-t.co.uk). As a portable and robust device, Diviner 2000 can be used to measure soil water over multiple depths (at 10 cm intervals) in the profile. With its probe and hand-held data logging display unit, onsite management decisions can be made at up to 99 sites (http://www.sentek.com.au).

Currently available Soil Atlases provide all information about soil sealing, erosion, organic matter loss, biodiversity decline, contamination, compaction, hydro-geological risks and salinization (http://eusoils.jrc.ec.europa.eu). GIS can be used to sample, test and localize precisely to evaluate soil fertility and nutrients. Soil fertility can be assessed accurately with GIS-assisted sampling, testing, and mapping. With grid or zone soil sampling, many soil properties, including macro- and micronutrients, pH, and salinity carbon content, can be tested.

### Crop canopy

Remote sensing techniques, such as spectroradiometrical reflectance, digital imagery, thermal images, near Infrared reflectance spectroscopy and infrared photography, provide tools for characterization of crop canopy. These tools can be used with airborne remote sensing platform to collect data for temperature, humidity, light, air, biomass and overage of the crop canopy. Robotic imaging platforms and computer vision-assisted analytical tools developed for high-throughput plant phenotyping (Fahlgren et al. 2015) can be used for measurement of the crop canopy. Automated recovery of three-dimensional models of plant shoots can be used for multiple color images (Pound et al. 2014). The 3-D structure can be also determined directly using laser scanning (Paulus et al. 2013) and deep time-flight sensor (Chéné et al. 2012).

### Companion organisms

Companion organisms are those surrounding crop plants, including bacteria, fungi, viruses, insects, weeds and even other intercropping plants (Fig. 1), which should be considered an important component of the environments. A series of methods and protocols have been developed to measure or determine companion organisms for different crops through multidisciplinary collaborations. For example, rhizospheric microorganisms can be extracted from bulked soil samples followed by comprehensive analysis and evaluation. Bulked sample analysis combined with metagenomics and DNA or RNA seq can be used to determine precisely the species, quantity, and mutual relationships of the organisms in bulked soil samples (Myrold et al. 2014). Using bulked samples collected from leaves or crop canopy, the organisms on the plant surface can be analyzed for their species, quantity, origin, distribution, developmental stages, and possible symbiontic relationships.

## Environmental characterization

**Fig. 2:**
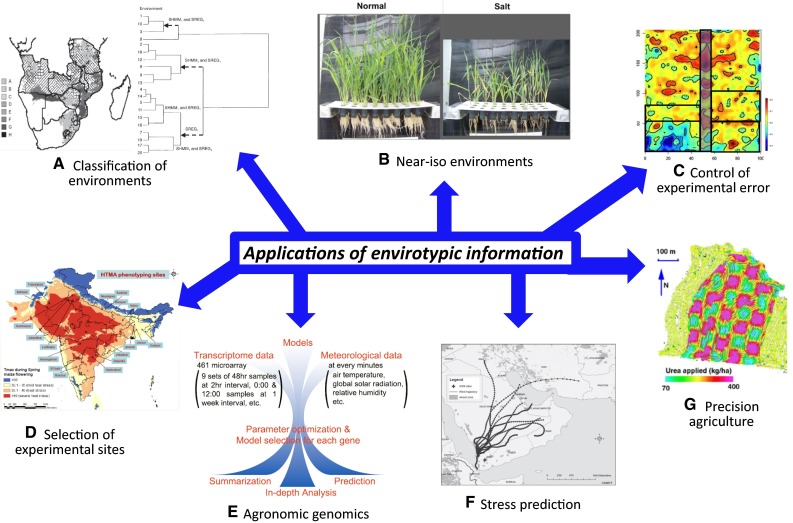
Applications of envirotypic information. Major applications include characterization of environments (**a** Bänziger et al. 2006; Crossa and Cornelius 2012), development of near-iso-environments (**b** http://www.google.com), control of experimental errors (**c** Prasanna et al. 2013), selection of experimental sites (**d** P. H. Zaidi, CIMMYT-India, personal comm.), agronomic genomics, studying the effects of crop management on gene expression (**e** Nagano et al. 2012), prediction of disease epidemics (**f** Singh et al. 2006), and precision crop production (**g** McBratney and Whelan 2001). Photos used for application illustration were selected from public websites or provided by CIMMYT colleagues, except for those indicated otherwise

Environments can be favorable and adverse to crop plants. The favorable environments are crop-friendly and resource-use efficient, while the adverse ones involve the pollutions and stresses of air, water and soil, and unfavorable climate changes. Environmental characterization is essential to experimental error control, data interpretation, data meta-analysis, and, in case of abiotic stresses, understanding patterns of resource availability (Fig. 2; Masuka et al. 2012; Trenberth et al. 2014). Envirotypic information can be used to reveal a series of important features for experimental and crop production environments (Xu 2015).

### Determination of field properties and within-site variability

Potential variables in a trial site can be largely reduced or eliminated while its historical features can be evaluated by environmental characterization. Soil mechanical impedance and depth can be measured by proximal sensors such as cone penetrometers. Due to the close relationship between ECa and clay, water, and ionic content, field gradients can be determined with electromagnetic surveys (Rebetzke et al. 2013; Gebbers and Adamchuk 2010). As the best indicator of field variability, crop performance, when combined with imaging techniques, wireless sensor networks and GIS, can be used to map and monitor spatial variability precisely (Lee et al. 2010). Linked with GPS, aerial high-throughput phenotyping platform enables fast non-destructive measurements of biomass. By conversion of biomass into normalized difference vegetation index (NDVI), such platforms provide a tool to measure field and within-experiment variability, which can be used to develop performance maps to guide next planting (Araus and Cairns 2014).

### Classification of environments

In mega-environment analysis, target environments can be classified into three types (Yan et al. 2007): single, simple mega-environments, which show no crossover genotype-by-environment interactions (GEI) with phenotypic performance repeatable across years; multiple mega-environments, which show crossover GEI that is repeatable across years; and single but complex mega-environments, which show crossover GEI that is not repeatable across years. Based on all available environmental information, potential trial sits can be accurately evaluated and thus the best trial sites can be selected. Such effort has been done in Africa for selection of the trial sites best suitable for stress tests of drought, low nitrogen, low pH, stemborer, and striga. The need for environmental data is particularly important in screening for drought tolerance where availability of soil moisture should be checked to ensure that the field condition and the drought stress imposed represent the target environment well (Römer et al. 2011). Three major criteria, maximum temperature, season precipitation and subsoil pH, have been used in sequential retrospective pattern analysis of environment similarity, by which eight maize mega-environments could be identified in southern Africa (Fig. 2a; Bänziger et al. 2006). Similarly, typical temporal modes of environmental variation for the soil–plant water balance have been identified for the US corn-belt target population of environments (Cooper et al. 2014).

### Construction of near-iso-environments

In many cases, experiments need to be done in two contrasting environments. The concept of near-iso-environments (NIEs), which is conceptually similar to near-iso-genic lines, was proposed to represent two contrasting environments that are significantly different in one major factor (Fig. 2; Xu 2002, 2010). One environment imposes much less stress on plants than the other. The less stress or normal environment can be used as control to measure the effect of the stress environment. A relative trait is then obtained from two direct traits phenotyped in the two environments to measure the plant sensitivity to the stress. If different plants show a similar phenotype under the less stress, the sensitivity can be measured using the direct trait value obtained in the more-stress environment. When both environments impose little stress the sensitivity should be measured instead using the relative trait value, that is, the difference of trait values measured in the NIEs, divided by the trait value measured in one of the NIEs or in the normal environment (Xu 2002). Traits suitable for measurement under NIEs include all abiotic/biotic stresses and plant responses to different environmental factors or crop management practices. NIEs can be used to facilitate identifying genes with environment-specific effects. In rice, days-to-heading (flowering) and photo-thermo-sensitivity were studied under conditions of field (Xu 2002) and greenhouse (Maheswaran et al. 2000), with different sets of quantitative trait loci (QTL) identified. Such QTL showing environment-specific effect have also been revealed for abiotic stresses under contrasting environments by integrative analysis of a large number of QTL reports (Des Marais et al. 2013).

### Control of environmental errors

Experimental errors are mainly contributed by micro-climate variability, non-uniformity of crop management and soil fertility, unpredictable influences of insects, diseases, weeds, companion microorganisms/plants/animals, winds, rainstorms and hails, and differences contributed by observation, measurement and production methods, tools, instruments, experimenters and farmers. Envriotyping can play a vital role in reducing environmental errors (Fig. 2). Experimental errors can be controlled through various approaches by reducing “signal-to-noise” ratio (Xu et al. 2012, 2013). (1) Trial sites selected should have uniform soil texture and fertility, relatively large in size, and fitting-in an appropriate rotation system, with good record of land utilization, representative of soil texture, climate, natural and economic conditions of the target environment. (2) Experimental materials under testing should be genetically homogenous with uniform individual samples (in terms of, e.g., seed quality and seedling age/size). (3) The common standards should be taken for all experiment managements and tests, with uniform application of resources and consistent control of weeds, pests and diseases. (4) Multiple replications, random treatments and controls should be included along with an appropriate experimental design. (5) Border effects should be minimized using boarder protect planting and selecting the trial sites far from villages, trees and highways. (6) New field-based techniques, such as precision resource application and remote sensing technology, should be used to measure secondary traits, by correctly selecting, calibrating and applying instruments, such as neutron probes, radiation sensors, and chlorophyll and photosynthesis meters (Xu et al. 2013). Climatic and soil moisture conditions can be characterized by wireless sensor networks, by which environmental conditions can be brought under real-time monitoring so that the environmental errors can be controlled (Araus and Cairns 2014).

Well-controlled or managed environments are often favored by molecular biologists because unwanted environmental variation can be minimized using pots, soil-filled pipes, hydroponics, growth chambers and greenhouses. However, it is still needed to achieve a better understanding of the environmental stresses prevailing under the nature conditions. Facilities for environment management become increasingly important, as they enable selection under controlled stresses (Rebetzke et al. 2013), improve crop performance measurement, and dissect phenotypic effects and their underlying genetic makeup (Blum 2011). However, controlled or managed environments could be very different from the nature or target environments (Masuka et al. 2012; Basu et al. 2015). Therefore, the results from controlled or managed environments could be far removed from what plants will experience in the field and will thus limit their application in germplasm development (Masuka et al. 2012; Araus and Cairns 2014). Compared to the field condition, for example, a pot is considerably smaller with a limited volume of soil available to roots, and the amount of water and nutrients will be limited to plants (Poorter et al. 2012; Reynolds et al. 2012; Basu et al. 2015).

## Crop modeling and phenotype prediction

### Models

As a third “typing” technology, envirotyping can be applied in many fields of crop science (Fig. 2). One of the applications is in crop modeling and phenotype prediction. With envirotypic effect as a new component, phenotype (*P*) can be partitioned into those contributed by genotypic effects (*G*), envirotypic effects (*E*), GEI (GE) and experimental error:

$$P \, = \, G \, + \, E \, + {\text{ GE }} + {\text{ error}}$$

With genotypic and envirotypic information available, *G* and *E* and thus the phenotype can be further partitioned. *E* can be partitioned into major components each consisting of several key environmental factors. In addition to crop management (*M*), both process and social economics can be also included as a part of *E*. For hybrid crops, phenotypic prediction can be performed for both inbreds and hybrids. Interactions of general and special combining abilities with *E*, GCA × *E* and SCA × *E*, can be estimated based on the responses of inbreds and hybrids to environmental factors.

Phenotype prediction can be performed with three modalities, i.e., new genotypes with known environments, known genotypes with new environments, and new genotypes with new environments (Bustos-Korts et al. 2016). Phenotype prediction can be simplified with consideration of co-variances and conditional variances. For example, prediction can be optimized using known soil properties and texture, etc., as co-variances, performed crop management as conditional variances, and improved simulation and modeling methodologies as facilitators. The prediction will be improved with managed conditions and precision envirotyping. If the contribution from *E* and GEI to phenotype is relatively small, the phenotype can be predicted largely by the genotype alone. Under well-managed environments, uniformity or good control of major environmental factors can be achieved and used to eliminate a large part of *E* and GEI, and thus they can be largely taken out of the prediction equation. On the other hand, when identical, homogeneous genotypes are phenotyped under natural conditions, the relevant *E* and GEI can be estimated. With some major environmental effects fixed, the rest *E* effects and thus the phenotype can be predicted. Near-iso-environments can be used to estimate the relevant major *E* and GEI. With the availability of known information about several major environmental factors for a target environment, a certain level of reliability can be achieved for prediction of important traits. For example, using over ten decades of actual data for maize yield and seasonal precipitation in Africa, a highly positive correlation between them has been established (CIMMYT, internal comm.), and thus the maize yield in Africa can be predicted largely based on the seasonal precipitation.

Phenotypic prediction needs to be conducted for individual traits and their conceptual models. In wheat, a conceptual model for yield and heat-adaptive traits has been developed (Cossani and Reynolds 2012):

$${\text{YIELD }} = {\text{ LI }} \times {\text{ RUE }} \times {\text{ HI}}$$

where LI is light interception which involves rapid ground cover and functional stay-green, RUE is radiation use efficiency, and HI is partitioning of total assimilates. RUE is dominated by three major components, photo-protection, efficient metabolism and water use. Photo-protection involves leaf morphology, down-regulation, pigment composition and antioxidants. Efficient metabolism is determined by CO_2_ fixation, canopy photosynthesis, spike photosynthesis and respiration. Water use efficiency involves the roots that match evaporative demand and regulation of transpiration. Partitioning (HI) consists of spike fertility, stress signaling, regulating, grain filling, and stem carbohydrate storage and remobilization (Reynolds et al. 2011). A similar model has also been constructed for yield under drought.

As one of the great efforts in phenotype prediction, the Genomes to Fields Initiative was established to predict traits from genotype and environment, thereby leading to improved maize production (http://www.genomes2fields.org). One of the subprojects, Genome by Environment, aims to assess environmental effects, using 31 inbreds and nearly 1000 hybrids tested in 22 environments across 14 states in the US and Canada. Similarly, Next-Generation Crop Breeding Platform for Predicting Germplasm Performance in Target Environments, proposed by University of Florida in collaboration with CG centers, is to develop a breeding platform for integrating and harmonizing genotype, phenotype, environment, and management data, and build next-generation crop-based models for predicting performance of genotypes in different environments (M. P. Reynolds 2015, presented at CIMMYT Science Week). However, greater efforts will be needed to establish a comprehensive predictive model to reveal the diverse biological networks, by which plants respond to combined climate change catastrophes. Such a model could be utilized to improve plant adaptation to changing climates (Ahuja et al. 2010).

### Genotype-by-environment interaction

GEI has been investigated recently through QTL mapping and gene cloning. QTL mapping using phenotypic data collected from multiple locations can be explored for understanding of the mechanisms involving GEI and their relative importance. The QTL with additive effects have four main GEI patterns (Des Marais et al. 2013): (a) antagonistic pleiotropy, with sign or direction changing of additive effects; (b) conditional neutrality with environment-dependent additive effects, which are limited to specific environmental conditions; (c) differential sensitivity, where the magnitude of additive effects are environment dependent; and (d) no GEI with no detectable change in additive effects across environments. One of the earliest GEI evaluations is to simply compare QTL identified across three locations in tomato (Paterson et al. 1991). QTL mapping under contrasting environments with one significantly different factor can be explored for understanding of actual GEI. Such studies are largely performed for abiotic stresses. From over 700 research reports, 37 of them with QTL mapped for abiotic stresses and complete QTL information available were selected for an integrative analysis (Des Marais et al. 2013), revealing that nearly 60 % of QTL exhibited GEI caused by antagonistic pleiotropy or environment-specific effects. These two GEI types showed strong influence on QTL effect plasticity as measured by the absolute difference in the standardized additive effects across environments.

Two types of GEI analyses have been done based on expression QTL (eQTL). One is based on the gene that shows different environment-dependent expression patterns in two genotypes. Another is based on genome-wide association study using expression levels of many genotypes across multiple environments. However, only few typical eQTL mapping reports are available with mapping populations tested under controlled abiotic conditions and quantified phenotypic expressions. One of the early formal eQTL studies involving an abiotic manipulation focused on *Brassica rapa* leaf tissue from plants grown under two levels of phosphorus (P) availability and identified over 3226 transcripts and several notable hot spots responsive to P, without formal tests of GEI (Hammond et al. 2011). Using an RIL population tested across soil drying treatments, thousands of genes that responded to soil drying and hundreds of main-effect eQTL were identified in Arabidopsis by eQTL mapping. However, very few eQTL were identified with significant interaction with the soil drying treatment (Lowry et al. 2013). There is accumulating evidence in model organisms such as yeast and flies that GEI are ubiquitous, accounting perhaps for the greater part of the phenotypic variation (Grishkevich and Yanai 2013). Such interactions appear to be caused by the changes to upstream regulators rather than local changes to promoters. Moreover, genes show different levels of GEI, and many factors, including promoter architecture, expression level, regulatory complexity and essentiality, are associated with the environment-induced differential gene regulation. For example, significant differences in response to drought or cold were found between the genes with consistent expression and the genes with variable expression under abiotic stresses. On average, the consistently expressed genes tended to share relatively more pairwise haplotypes, with lower promoter diversity and fewer nonsynonymous polymorphisms (Lasky et al. 2014).

To understand the origin, spread, and evolutionary processes of GEI, the specific genes that control GEI phenotypes and the mutational variants that define functionally distinct alleles should be identified (Des Marais et al. 2013). We need to determine which of the following factors are more often GEI driver in plant abiotic stresses: gene type (e.g., environmental sensors, biosynthetic enzymes, or regulatory proteins), gene characteristics (e.g., paralogs, or complex cis-regulatory control), mutation type (coding/noncoding, or transposable elements, etc.), and molecular mechanisms (condition-dependent epistasis, gene expression, enzymatic activity). Genes exhibiting GEI can be identified and used in comparative analyses across lineages and tests for parallel and convergent evolution in responses to the environment. The complex regulatory systems plants have evolved to control phenology are driven largely by environmental cues such as photoperiod, temperature, and circadian signals (Kim et al. 2009). As summarized for cloned GEI genes using flowering time and soil and water availability as examples, a variety of natural variants and mechanisms have been revealed at the molecular level, including nonsynonymous changes in receptor proteins, loss-of-function mutations in transcriptional repressors, splicing variants in biosynthetic enzymes, and gene duplication in transcription factors (Des Marais et al. 2013).

To predict GEI, environmental covariates and crop modeling have been integrated recently into the genomic selection framework through factorial regression model (Heslot et al. 2014). Stress covariates for predicted crop development stages were derived from daily weather data, with model tested by a large wheat dataset. For unobserved environments with available weather data, the accuracy of genotype performance (phenotype) prediction increased by 11.1 % on average. With insight into the genetic architecture of GEI provided by this model, genotype performance could be predicted based on available environment data such as past and future weather scenarios. In another report, using covariance functions GEI was modeled through interactions between high-dimensional sets of markers and environmental covariates (Jarquín et al. 2014). Using data from 139 wheat lines genotyped by 2395 SNPs and phenotyped for grain yield over 8 years across locations in northern France, prediction accuracy substantially increased (17–34 %) by including interaction terms in the models compared to models with main effects only. Similarly, GEI was modeled in genomic selection using a marker × environment interaction (Lopez-Cruz et al. 2015), which was used to analyze three CIMMYT wheat datasets with over 1000 lines genotyped by GBS and phenotyped at CIMMYT. The model had substantially greater prediction accuracy, compared to an across-environment analysis with GEI excluded.

So far, environmental information has been used, collectively in almost all cases, as a component in the model to investigate GEI and the phenotypic performance across different environments, without partitioning into individual environmental factors. Such evaluation usually does not involve any envirotypic information, by assuming that different locations have different environmental effects and thus difference revealed in genetic and molecular analysis can be attributed to GEI. With more thorough understanding of genotypic information, we can now precisely describe genes, alleles, haplotypes and their integrative contribution to a phenotype. As a result of envirotyping, we should be able to dissect the *E* component into individual factors. By incorporating precise measurements of *G* and *E* with precision phenotyping, therefore, GEI can be evaluated precisely, and predicted based on the theoretical model established with *G* and *E* information.

### Environment-responsive genes

Plastic responses to environmental signals can occur at the molecular level. To initiate a signaling cascade, a receptor at the cell surface must first perceive an external stimulus. The environment-responsive genes can be classified into two major categories: one responding to neutral environments such as photoperiods, regular temperatures and normal nutrient levels, and the other to the environments with abiotic stresses such as drought, waterlogging, extreme temperatures and deficiency of essential nutrients. Under half of the studies Alvarez et al. (2015) reviewed (41 %) addressed how gene expression can be affected by environmental stimuli, such as abiotic stress, environmental heterogeneity in time or space, host–parasite interactions and potentially selective biotic and abiotic interactions. Ten years of transcriptomics in natural environmental fluctuations have shown that stress responses can have significant impacts on many categories of genes, and transcription may be affected by even small environmental changes. Responses to the environmental change can involve the post-translational modifications of the components of signaling pathways (Nühse et al. 2007). Alternatively, regulatory gene transcription can respond to a wide range of external stimuli. Gene expression alternation and thereby plasticity generation can be created by epigenetic processes, such as DNA methylation, histone modification and transposable element activation (Chinnusamy and Zhu 2009). Variation in small RNA populations can lead to post-transcriptional control (RNAi) as well as changes in chromatin modification. Lastly, gene expression can be also affected by the expansion of short repeat sequences (Nicotra et al. 2010). With large-scale epigenomic analysis involving large numbers of genetic samples, spatial and temporal effects on families or inbreds can be partitioned to optimize the genetic variation to identify frequent epialleles.

Under well-managed or near-iso-environments, genes responsive to climate change catastrophes can be dissected. It is important to decipher and predict plant dynamics under field or natural conditions (Izawa 2015). Many genes have been cloned with function analyses for their responses to major neutral or extreme stressful environments. Identification of specific genetic determinants of stress adaptation to waterlogging, drought, low temperature, Al toxicity and salinity has revealed that the genetic loci are often associated with distinct regulation or function, duplication and/or neofunctionalization of genes that maintain plant homeostasis (Mickelbart et al. 2015). At the same time, a large number of genes have been cloned for biotic stress tolerance, including over ten genes for rice blast resistance (as summarized in Chen et al. 2015). In addition, a series of genes responsive to neutral or normal environmental factors, such as normal light and temperature (for photo- and thermo-sensitivity), have been cloned (as summarized in Matsubara et al. 2014).

Due to selection of dramatic fluctuation of diverse environmental factors, wild plants have evolved with their genetic networks responsive to such complex nature conditions. Plant adaptation to environmental stresses are coordinated and fine-tuned by adjusting growth, development and cellular and molecular activities. Responses to stresses are usually accompanied by major changes in the levels of transcriptome, proteome and metabolome. The metabolic adaptations to environmental stress factors involve increase, decrease or accumulation of various metabolites in leaves, shoots, roots, flowers, seedlings, grains, and nodules of plants (Ahuja et al. 2010). As an important contributor to abiotic stresses, miR156 isoforms are highly induced by heat shock, and the miR156-SPL module mediates the response to recurring heat shock in *Arabidopsis thaliana* and thus may function to integrate stress responses with development (Stief et al. 2014). Plant adaptation to environmental stresses is modulated by a myriad of genes, proteins and metabolites, and their corresponding metabolic pathways or biological networks. Phenotypic expression over both space and time is inconsistently affected by environmental variability, which should be accounted for any statistical models for estimation of parameters of interest (Cobb et al. 2013; Araus and Cairns 2014). Further identification of the genome architecture associated with responses to particular stimuli might help us predict plastic responses to adverse environments imposed by climate change (Nicotra et al. 2010).

### Prediction of biotic and abiotic stresses

Based on the environmental factors and their predominant changes that affect disease and pest epidemics, epidemic time, place and distribution of biotic stresses can be predicted, in combination with other relevant social factors (Fig. 2). Such prediction can be also done to forecast new abiotic stresses caused by significant weather variation and climate change. In general, biotic and abiotic stresses can be predicted based on Gb (genotype of biofactors in case of biotic stresses), Gh (genotypes of the host crop), *E* and their interactions. *E* should include all the environmental factors that affect crop growth and yield, dominate the boom, bust and epidemics of diseases and pests, and impose abiotic stresses to crop plants.

Prediction of biotic stresses involves examining new diseases and races due to weather variation and climate change, establishing prediction model to forecast new diseases and races, and their spread paths and speeds. The prediction should be done for major environments, major experimental stations and production zones. With detailed envirotypic information available, prediction may be done for experimental blocks, plots and even individual plants. To predict abiotic stresses, environments should be well characterized, particularly for the prevalent factors.

Biotic stresses can be predicted for both short and long terms. The former is important for farmers to take actions to reduce stress-related losses. Such prediction should be done largely for the coming season or year based on currently available and forecasting weather data, in combination with historical data on diseases, pests and climate. With the prediction, forecasting before the season, boom or bust cycle is highly preferred. The long-term prediction is important for scientists to develop techniques and varieties to be prepared for the diseases and pests to change, evolve, or move. It is largely done for the coming years and for their movement and spread, based on historical and current climate data and foreseeable climate change with updating knowledge on the epidemic diseases/pests, host–pathogen interaction (GbXGh), GEIs (GhXE, GbXE, and GhXGbXE), and the genes and genotypes of the host plants against diseases and pests. Based on a large number of pests and pathogens examined, an average poleward shift of 2.7 km per year since 1960 has been demonstrated, while a significant variation in trends was detected among taxonomic groups (Bebber et al. 2013). The positive latitudinal trends observed in many taxa provide evidence to support the hypothesis that global warming has driven the pest movement.

Wheat rust Ug99, detected first in East Africa, is one of the best examples for disease prediction. Relevant factors that determine movement of Ug99 in wheat have been integrated to predict the rust epidemics (Singh et al. 2006). The factors include the current status and distribution of the rust, prevailing winds, climatic factors favoring survival and sporulation, wheat production zones (geographical distribution and associated human populations), historical migration patterns for the rust races with East African origin, and responses of existing cultivars to the rust. The rust monitoring can be established and optimized by developing standardized data collection, building up lab capacity for rapid diagnostics, centralizing data management and information dissemination, monitoring both pathogen and host in an integrative way, and establishing early warning/forecasting system.

Prediction for abiotic stresses should be more straightforward as the relevant environments are the direct causal factors. Stressful factors may come from climate (radiation, temperature, precipitation, air, etc.), soil (nutrients, moisture, pH, salinity) and water (Deinlein et al. 2014; Lobell et al. 2014; López-Arredondo et al. 2014; Hu and Xiong 2014). Abiotic stresses caused by soil factors, modified largely by production activities, are more predictable and measurable than biotic stresses. Climate factors can be largely predicted based on latitude, longitude and elevation. Compared to climate changes that cause long-term variation, weather changes cause short-term, less-predictable fluctuations. Both climate and weather changes may cause significant variation of abiotic factors and thus stresses on crop plants.

It has been predicted that global climate change will have significant impacts on crop productivity by creating significant abiotic stresses on crop plants such as ozone and heat. Depending on production zones, some crops show primary sensitivity to single stresses such as ozone (e.g., wheat) and heat (e.g., maize) (Tai et al. 2014). High temperatures contribute to reduced crop yields, and predicted global warming has raised growing concern regarding future crop productivity and food security. Without adaptation, losses in crop production are expected for three major cereals (wheat, rice and maize) in both temperate and tropical production zones by 2 °C of local warming (Challinor et al. 2014). Yield gains in most wheat-growing regions have been slowing down by global warming, and global wheat production would become more variable over locations and time, with production decrease by 6 % for each °C of further temperature increase (Asseng et al. 2015). With climate change, more frequent adverse weather conditions will happen in European wheat zones. For example, adverse conditions for 14 representative European wheat sites might substantially increase by 2060 compared to 1981–2010, with more frequent crop failure expected (Trnka et al. 2014). In maize, when adaptation is accounted for, average yield losses in the US from a 2 °C warming would be reduced from 14 % to only 6 % (Butler and Huybers 2013). Rainfed maize yields in the US (Schlenker and Roberts 2009; Lobell et al. 2013) and elsewhere (Lobell et al. 2011) have indicated a strong negative yield response to accumulation of temperatures above 30 °C. Maize also shows increased yield sensitivity to drought stress caused by high vapor pressure deficits. Translation of improved drought tolerance into higher average yields becomes more acceptable agronomic changes than decreasing yield sensitivity to drought at the field scale (Lobell et al. 2014).

As abiotic and biotic stress combination becomes very common, it is essential to predict all relevant stresses simultaneously. Responses to combined stresses are genetically controlled to a great extent by different, even functionally opposing, signaling pathways that may interact and inhibit each other, and therefore, it would be impossible to extrapolate directly the response to simultaneous multiple stresses from those to single stresses (Suzuki et al. 2014; Prasch and Sonnewald 2015; Ramegowda and Senthil-Kumar 2015). By omics and functional analyses of individual genes, a convergence of signaling pathways has been revealed for abiotic and biotic stress adaptation. Complicated potential effects of abiotic stress have been expected on resistance components, for example, extracellular receptor proteins, R-genes and systemic acquired resistance. Such elaborated stress resistance crosstalk would also happen at the levels of hormone, reactive oxygen species, and redox signaling (Kissoudis et al. 2014). The prediction for possible combined stresses would help develop breeding strategies for manipulation of individual common regulators and pyramiding of non-interacting components.

### Integration of phenotyping with envirotyping

Crop modeling and phenotype prediction depend on precision phenotyping as a feedback correct for the best model-fitting. A new trend in crop science will be to combine high-throughput precision phenotyping with large-scale envirotyping to collect, mine and utilize the two sets of information comprehensively. Currently, many robotic, high-throughput phenotyping systems are built under controlled or well-managed environments. Automatic phenotyping platform has been established for plants in growth chambers with major environmental factors controlled (Jansen et al. 2009; Massonnet et al. 2010). Expensive sensors used in phenotying can be either in fixed location with moving plants, in mobile device with fixed plants, or colocalized with plants. However, it is very challenging to combine the high-throughput phenotyping with large-scale envirotyping for several reasons. First, there are a large number of genotypes to be tested under a wide range of environments. Second, precision phenotyping needs to be done across most, if not all, of the developmental stages and spaces. Third, it is very expensive. Due to the cost and capability limitation, early phenotyping efforts are seldom close to meeting the requirements of complete phenomics (Houle et al. 2010). Probably, more challenges come from establishing such high-throughput phenotyping platforms for the crops with big plants and long life cycle such as maize and sorghum, because it is very difficult to build up large enough controlled environments for a large number of big plants to grow for a long time.

Precision phenotyping needs to be coupled with precision envirotyping, as envirotyping plays a vital role in generating phenotypic data of high quality, consequently, improving crop research. Field variation in soil, moisture and fertility contributes to error variances, thereby masking major genetic variation for important traits and reducing repeatability, regardless of the cost and precision of available phenotyping platforms (Masuka et al. 2012). High-throughput platforms allow phenotyping a huge number of genotypes growing in a larger field, thereby increasing soil variability. In general, the larger the land is required for an experiment, the harder it becomes to identify a land with minimum soil variability (Araus and Cairns 2014).

Phenotypic and envirotypic information collected for the same set of genotypes will greatly contribute to crop modeling and phenotype prediction by complementary and comparison analyses. Firstly, phenotypes collected for homogeneous, identical genotypes under large-scale experiments can be used to reveal within-site variation and environmental variability, because significant phenotypic variation can be attributed to significant environmental variation and GEI. Secondly, under well-managed environments with appropriate experimental error control, phenotypic variation in genetically different materials can be largely attributed to genetic contribution. Thirdly, under near-iso-environments, or controlled vs uncontrolled environments, phenotypic differences are the genotypes’ response to the major environment factor. Fourthly, envirotyping of all environmental factors along with phenotyping will reveal integrative phenotypes for different genotypes, by which we can determine the contribution of genotypes and their interaction to the phenotype. Fifthly, phenotyping overtime using homogenous genotypes will reveal dynamic environmental variation. A recent integrated phenotyping with soil content in maize rooting zones indicates that under water-limited conditions grain yield increased significantly without significant increase in total water extraction (Reyes et al. 2016). Therefore, the measured long-term genetic gain for yield must have been achieved through improved maize adaptation to water stress conditions by either increased water use efficiency or increased carbon partitioning to the grain.

### Evirotyping for single plots and individual plants

**Fig. 3:**
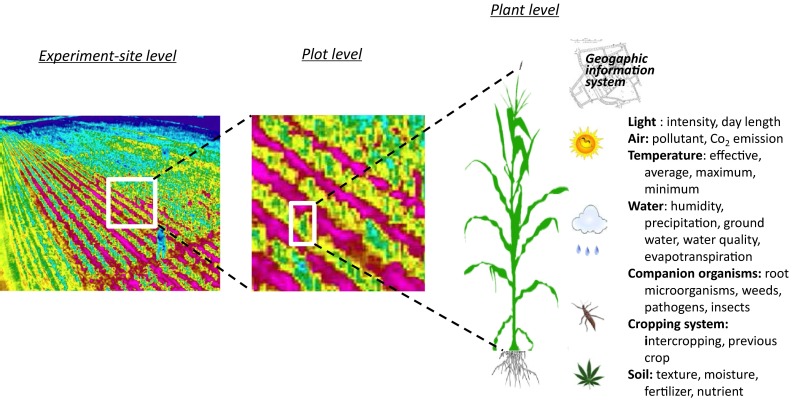
A zooming-in process of envirotyping. It is vital to move envirotyping from the levels of experimental stations and the whole field blocks to individual experimental plots and plants by a zooming-in process so that envirotypic information collected can be matched up with genotypic and phenotypic data for each entry or target plant. Revised from Xu (2015)

For some major environment factors, such as soil moisture, nutrients and pH, we can now implement envirotyping at the level of individual plots, single rows, or even single plants. For most environmental factors, however, it is not possible for a real envirotyping until some great technical innovations have been achieved. In one hand, variation for some environmental factors might be too small to be detectable, and thus facilities currently available need to be improved significantly in terms of sensitivity, precision and resolution. On the other hand, envirotyping process with well-equipped facilities may significantly disturb or interfere with plant growth and development, resulting in significant micro-environment changes around the crop plants. It can be expected that technical innovations with increased precision and minimized disturbance on crop plants will allow our current environmental data collection move from the level of experimental station to the whole block and then to individual plots and single plants, as a zooming-in process of photographing to focus on specific details with high resolution (Fig. 3). By the end, we can generate plot- or plant-based envirotypic data to match up with genotypic and phenotypic data for each genotype.

The bottleneck for a plot- or plant-based envirotyping could be the high cost involved along with a data tsunami. Significant advances in development of platforms and facilities are essential for envirotyping to match the current scale and resolution of genotyping and phenotyping, since crop improvement and production involves a large number of varieties (genotypes) each involving a huge number of plants. With such a numbers game, significant cost reduction will be needed, including much cheaper high-throughout and precision envirotyping facilities, less labor-intensive processes, and highly effective information management platform.

In additional to the technical difficulties and potential high cost, dynamic envirotyping across development stages and individual plots/plants will result in a data tsunami that might be more difficult to manage compared to genotypic and phenotypic data, as envirotyping data formats vary greatly with huge numbers of images and videos generated across developmental stages. Moreover, understanding the effect of any single environmental factor often means that we have to control the rest, which might be difficult for some environmental factors. On the other hand, standardization of environmental designs for single environmental factors, which is required for cross-research and cross-lab comparisons, needs to consider the masking effects of one major environmental factor over the others.

## Envirotyping-driven precision agriculture and breeding

### Precision agriculture

Agricultural activities have accumulated many types of data, which associate with climate, soil, disease and pest epidemics, relationship between yield and plant density, and market prices of agricultural products. Data-based integration, modeling and simulation help make decisions on large-scale agricultural production. In industrial countries, modern agriculture is moving from mechanization to informatics, particularly, envirotyping driven, allowing high precision and efficient breeding and crop production (Fig. 2). As a farming management concept, precision agriculture is developed based on observing, measuring and responding to field variability, weather conditions and other external environmental factors. More generally, it should include climate-smart agriculture and intensification involving irrigation, fertilization, cropping system and intercropping. Crop performance varies typically with both space and time which involve statistical treatments. The holy grail of research in precision agriculture will be the ability to develop information management and decision support systems for functional farm management to optimize returns on inputs while preserve resources.

Multi-national corporations have been investing heavily to establish a research and development system with decisions supported by big data (Cooper et al. 2016). To do that the seed business giant Monsanto recently purchased two companies, Precision Planting and Climate Corporation. Under guidance of these two companies, samples are collected for soil tests from each point of every 4-acre of land, providing detailed soil information for famers to make decisions on fertilization, irrigation and also to predict the yield that can be achieved for different crops. Through its climate software, Climate Corporation provides farmers with farm-wide real-time weather data, including temperature, humidity, wind, precipitation, which can be used to determine when planting, harvesting and crop management should be done for a given block of the farm land.

Using mobile systems supported by big data, real-time soil moisture, temperature and crop growth status can be obtained, so that farming precision can be significantly improved. Precision planting manufactured precision agriculture equipment that can be fixed in powerful tractors, parallel running planters or other machines. With built-in application software, decisions can be made on when the crop field should be checked and when pesticides and fertilizers should be applied. Real-time soil moisture can be collected and used to make irrigation decisions. Such real-time monitoring has become increasingly important with increased climatic variability, particularly during the off-season and in managed drought phenotyping (Araus and Cairns 2014). For precision farming supported by big data, all agricultural inputs can be brought under precise control so that energy, fertilizers, water and pesticides can be significantly saved. Through GPS, auto-driving system, computer facilities and essential sensors, the information provided by big data software can be accessed, manipulated and transferred to realize intelligent agricultural mechanization. According to soil properties, such intelligent systems can adjust their planting to make the seed sowed at the same depth. They can also improve the operation quality, for example, by increasing the ratio of single seed planting to 99 %.

With historic climate data, Climate Corporation can provide more accurate weather prediction for a small area of land. The basic model is to develop an informative farm map using global germplasm information, historic yield data and Climate Corporation’s climate data, and then put all possible climate information onto the map. With all the data support, the company can provide farmers with crop insurance service, and the farmers can access to the map to search for specific information and to determine which crops and crop varieties should be planted and under what conditions they have a good harvest.

In the public sector, landscape-scale crop assessment tool (LCAT), a cloud-based tool developed by CIMMYT scientists in collaboration with Oak Ridge National Laboratory and GEOGLAM (University of Maryland), can be used for crop land identification, crop classification, phenology test, crop status measurement, and in season-forecasting input. Data sources include those from satellites (Landsat, Aster, MODIS, VIR, etc.), satellite-derived soil moisture and other products, and weather (Urs Schulthess, CIMMYT, personal comm.). As an example of applications, N-rate can be calculated by GreenSat for a crop field using NDVI maps derived from SPOT satellite with N-rich strips identified. Within-field (or rather treatment) variability can be detected, as ground cover is measured directly by the amount of sun light that a crop captures for photosynthesis (Ortiz-Monasterio et al. 2013). Actionable advice on crop management, planning, decisions and field operations can reach farmers through mobile phones to guide nutrient management (sources, rates, and times for field size, and N timing adjusted for water availability), crop establishment (seed source and seed rate), crop protection (weed, disease and insect management), irrigation (amount, time and frequency), and yield estimation (before and at harvest) (Urs Schulthess, CIMMYT, personal comm.).

### Agronomic genomics and improvement of companion organisms

To better understand the effects of agronomic practices on crop growth and yield, the genomic approaches widely used in genetics and crop improvement should be explored for agronomy. Agronomic genomics aims to integrate agronomy with omics to develop a high-efficient, cost-effective, and environment-friendly crop management system to optimize the gene expression and thus the crop production (Fig. 2). Effects of agronomic practices, such as fertilization, irrigation, pest/disease management, weed control, etc., can be revealed by the changes of the level and pattern of gene expression as revealed by DNA-, RNA- and protein-sequencing technologies. Dynamics of gene expression patterns may be directly determined under complex and changing environments. Currently, environmental factors that shape transcriptomics are complex, vulnerable and multiplexed, making it difficult to integrate all the data collected for crop management for meaningful data mining. Nevertheless, agronomic genomics will facilitate breeding crop varieties with improved responses to crop management.

Compared to the natural conditions where crop plants experience, complicate environmental changes across their growth and developmental stages, i.e., the dynamics of gene expression changes, can be determined more directly and accurately under managed or controlled environments. Using transcriptome data collected from rice leaves along with the meteorological data in the field, including wind intensity, air temperature, relative humidity, atmospheric pressure, global solar radiation and precipitation, statistical models were developed for the endogenous and external influences on gene expression (Nagano et al. 2012; Fig. 2). The transcriptome dynamics is revealed to be predominantly governed by several key factors such as endogenous diurnal rhythms, ambient temperature, plant age, and solar radiation. Diurnal gates for environmental stimuli affected transcription and pointed to relative influences on different metabolic genes exerted by circadian and environmental factors. In Arabidopsis, DNA microarrays were used to reveal how gene expression changes with development and responds to environmental conditions (Richards et al. 2012). Differences in accession and developmental status could equally explain the variation in gene expression in two accessions of *A. thaliana* grown in field conditions, and gene expression was significantly predicted with temperature and precipitation. Using a relatively simple design and several environmental factors, these two studies identified the molecular basis of response to environmental changes and teased apart the influences of development and complex environmental variables (Nagano et al. 2012; Richards et al. 2012), and should be applicable to other crops for deciphering the impacts of complex environments on transcriptome fluctuations.

Companion organisms exist on or around the plants, particularly in the rhizosphere. Crop yield, quality and stress tolerance are largely affected by soil environments, particularly the rhizosphere microorganism community. Therefore, simultaneous improvement of crop and its rhizosphere microorganisms has significant implications in genetics, ecology and agronomy. Improvement of the rhizosphere microorganisms can help establish better environmental conditions for crop plants. The improvement involves inorganic and organic conditions. The former includes upgrading of water and fertility maintainability of the soil, transfer of ineffective inorganic nutrients to effective ones, and degrading of soil toxic matters. The latter involves inhibiting unfavorable soil organisms but enhancing favorable ones. Improving rhizosphere microorganisms will play roles in increasing crop yield similar to improving abiotic and biotic stresses of crop plants per se. Therefore, companion organisms should be improved under the guidance of envirotyping. Molecular biology can be used to modify and optimize the environments surrounding the crop plants, and it is important to transform crop improvement from the crop oriented to crop community oriented.

### Four-dimensional profile of crop breeding

**Fig. 4:**
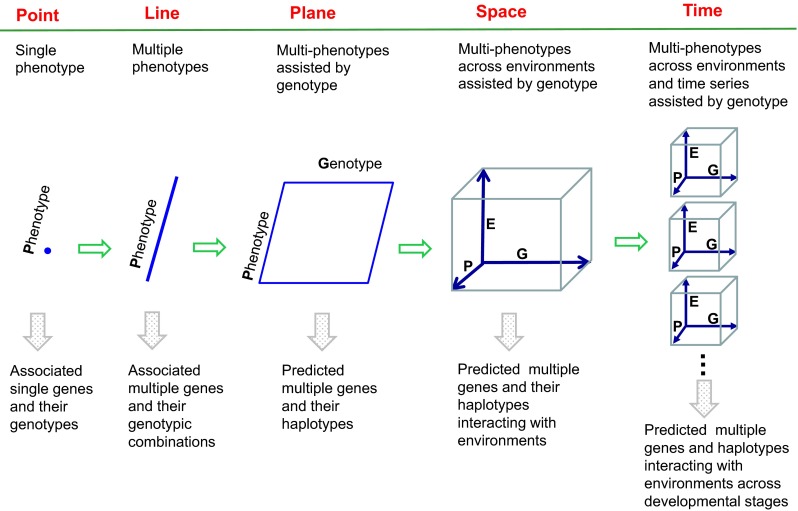
A four-dimensional profile of precision breeding and crop production system with the concept evolving from point to line, plane and space. Selection in early plant breeding was performed based on single desirable phenotypes one at a time (‘‘point’’). Conventional breeding has been based on selection of multiple phenotypes (‘‘line’’). Marker-assisted breeding uses selection criteria determined by both multiple phenotypes and genotypes (‘‘plane’’). Our future breeding and crop production system will be built upon the knowledge generated by genotyping, phenotyping and envirotyping, which forms the three spatial dimensions (‘‘space’’). Considering the temporal variation across different growth and developmental stages, a fourth dimension (time) should be also included. Green arrows represent the evolutionary steps of plant breeding; gray-dotted arrows represent the selection targets that can be inferred from selection strategies. Revised from Xu et al. (2012)

Environmental information has not been well exploited for improving our understanding of plant adaptation. It is being complemented with environmental characterization through large-scale envirotyping via information systems such as GIS. It can be exploited for plant breeding and crop production in various ways, including but not limited to, precision measurement of environmental factors affecting specific developmental processes, selection of target environments for specific experiments, designation of environmental factors associated with phenotypic variation, dissection of GEI factors into specific components, and breeding for improved response or adaptation to specific environments, environmental factors and their combinations. Ultimately, an optimized precision breeding and crop production system can be built up with a four-dimensional (4D) profile, the first three (3D) being spatial determined by genotype, phenotype and envirotype, while the fourth being temporal involving developmental stages (Fig. 4), and thus a cultivar or genotype architecture can be designed with an optimized phenotype and best adaptation to a target environment or crop management system. With genome-wide understanding of the environmental impacts on crop plants—enviromics and long-term trial data and outputs from general circulation models to form climate change-oriented breeding—breeding by design can be driven by incorporating information from genotyping, phenotyping, and envirotyping across developmental stages. With the support of the 3D (*G*–*P*–*E*) information, for example, management of wheat rust race Ug99 can facilitate establishing more effective *G*–*P*–*E* models for biotic stresses (Dave Hudson, CIMMYT, personal comm.). Monitoring systems for tackling biotic stresses that have been established with *G*–*P*–*E* informatics are well in the position to predict temporal change of the relevant stresses for a 4D informatics-driven precision breeding.

“Sustainable Intensification” aims at increasing crop production through more efficient use of all resources, while minimizing pressure on the environment and developing resilience, natural capital and environmental services. Breeding under conservation agricultural system can help understand if full selection under conservation agriculture conditions results in genotypes with better performance under such conditions, better emergence vigor/crop establishment, and better performance under water-limited conditions. It may also help us understand if such a full selection would result in genotypes with excessive height and tendency to lodge, and less earliness. However, little has been done through breeding to fully realize the yield potential of new germplasm, for example, under no-tillage. On the other hand, low GEI (15 % of variability) suggests that no separate breeding program would be required for conservation agriculture (Hlatywayo et al., Cairns and Thierfelder; CIMMYT, personal comm.). As envirotypic information becomes available for all the factors under conservation agriculture conditions, a precision breeding program can be designed and optimized based on the 4D breeding profile so that the breeders can deal with complexity without being lost in complexity (Fig. 4).

The concept of 4D plant breeding profile can be used to identify the best environments for a specific crop and to adjust breeding strategies for the changing target environments. As one of the examples for movement of crop belts, U.S maize production increase from 1.8 to 12.7 billion bushels during 1879–2007 accompanied a substantial change of the footprint of production (Beddow and Pardey 2015). During this period, the US corn growing areas moved 279 km north and 342 km west. The new spatial output indices developed showed that such spatial movement contributed to 16–21 % of the yield increase in US maize production over the 128 years. This long-run perspective provides historical precedent for how much crop production might adjust to future climate change and technology innovation.

### Information and support systems

The phenotype we observed is the outcome of the interactions between constantly changing environmental factors and a certain genotype. A full understanding of this process posts a great challenge and depends on high-throughput envirotyping platforms and the power of computation to analyze big datasets. In addition to currently available large datasets created by genotyping and phenotyping, envirotyping will generate a huge amount of information including images and videos, causing a data tsunami in the near future. Therefore, the *G*–*P*–*E* data space should be expanded to include a fourth dimension contributed with the time (*T*) that lasts through the whole developmental stages of crop plants. There are 148,000 wheat accessions stored in CIMMYT Genebank and 113,000 rice accessions in IRRI Genebank. These accessions can be genotyped using SNP chips to produce thousands to millions of genotypic data points per accession, or sequenced to produce Tb levels of DNA, RNA and protein data. To meet the challenges of increasing envirotypic information and to prevent agronomists and biologists from devouring by the data tsunami, standardized data generation and collection procedures, data collecting tools, sampling technologies, controlled vocabularies and ontologies, and interoperable query systems should be established. An integrative information system will need a general database with ontology control to bring genotypic, phenotypic and envirotypic information together, which is particularly important for comparison and integration across crop species. In general, we need to develop a generic database and information system for all the data from different *G*–*P*–*E*–*T* sources. As significant efforts have been made in development of controlled vocabularies and ontologies for genotyping and phenotyping (Xu 2010), we need to follow the scenarios to develop the similar system for envirotyping. Unique identifiers that are associated with each concept in envirotyping should be developed and used for linking and querying databases.

High-performance computing cluster, hardware and software are needed for data analysis. Considering that *G*–*P*–*E*–*T* data have been generated and maintained largely through different research programs and groups, it is vital to establish a one-step-shopping system to allow all the data accessible to all relevant scientists and users. Systems biology approaches driven by information could prove beneficial and biological models could be generated finally to show the contribution of different signaling pathways through building plant ‘-omic’ architectural responses to climate change catastrophes (Ahuja et al. 2010).

To meet the data tsunami challenge, a successful crop science research program needs to back up with appropriate decision support tools. Integrative Breeding Platform (IBP) provides tools to help breeders in designing and managing experiments, collecting and storing data, and conducting analyses (https://www.integratedbreeding.net). Its interconnected software, Breeding Management System (BMS), is designed for breeders to manage their daily activities throughout their breeding programs. However, the databases and tools currently available or under development are largely for genotypic and phenotypic information, with little consideration of environmental data. Apparently, incorporation of all environmental information into databases and tools deserves a special attention. Future changes for long-term breeding program orientation can be predicted by utilizing downscaled general circulation model outputs in combination with mega-environment definitions, crop production areas, specific areas or locations, and socioeconomic data. Learning from the best and the past through data mining will help us to develop an integrated approach to optimize crop production.

## Future prospects

In the future, envirotyping will face more challenges than what genotyping and phenotyping have ever. Human resources and investments are needed to be dispatched among genotyping, phenotyping and envirotyping for a balanced development. As high-throughput and sequence-based genotyping becomes routine and high-throughput and precision phenotyping becomes achievable, a full set of next-generation high-throughput precision envirotyping technologies will be also showing up at the corner. As in many cases, where biologists present challenges while computational scientists present solutions, envirotyping tools and methodologies need to be developed through multidisciplinary collaborations by standing on the shoulder of history. Companion organisms have more complicated interactions with host plants than other environmental factors. Predictive phenomics would become possible with more and more envirotypic information available. Understanding the effects of crop management on gene expression will help us design more cost-effective, sustainable and multifunctional crop production systems. Envirotyping will help identify the best sets of crop management practices to optimize yield and yield components such as plant density. A great contribution to breeding and crop production by envirotyping would be the precision agriculture largely driven by envirotypic information and reducing drudgery. Due to increasing soil and water pollutions, demanding for healthier and nutritious food, which is becoming increasingly important in developing countries, will drive to establish soil–human health relationship and to develop new tools and methodologies for dynamic and continuous envirotyping to monitor the entire environmental profile. Cutting childhood mortality in half (FAO 2008) through biofortification by 2050 means that we need to develop new varieties with improved nutrition by combining varietal improvement for the changing soil nutrients. Envirotyping-guided crop science will help us to face all the challenges through genetic studies, genotype reconstruction, variety development, abiotic/biotic stress prediction, and precision agriculture and breeding. Envirotyping, as a third “typing” technology for crop science, should be used to decipher all environmental impacts on growth, reproduction and survival of crop plants. With large-scale envirotyping, in combination with known genotypic information, environments for crops can be optimized and phenotypic performance under specific environments can be largely predicted, thus enhanced cost–benefit efficiency for precision breeding and crop production.
